# Biofilm structure as a key factor in antibiotic tolerance: insights from *Bacillus subtilis* model systems

**DOI:** 10.1038/s41522-025-00864-x

**Published:** 2025-12-18

**Authors:** Mojca Blaznik, Marko Volk, Barbara Kraigher, Alba Calonge-Sanz, Gema Barco-García, David Stopar, Iztok Dogsa

**Affiliations:** Department of Microbiology, Biotechnical Faculty, University of Ljubljana, Ljubljana, Slovenia

**Keywords:** Microbiology, Antimicrobials, Biofilms

## Abstract

Tolerance to antimicrobial agents in mature and structured biofilms presents a significant challenge in clinical and industrial applications. The contribution of biofilm physical structure to antimicrobial tolerance remains particularly poorly understood, primarily due to the lack of biofilm structure quantification and manipulation studies. To fill the gap in our knowledge, we have investigated how mechanical and biochemical disruptions of biofilm integrity affect *Bacillus subtilis* tolerance to antimicrobial agents. Our findings reveal that biofilm structural integrity is a major determinant of tolerance to membrane disrupting antibiotic daptomycin. Biofilm viscoelastic properties as well as antimicrobial tolerance to daptomycin were directly related to the presence of exopolysaccharide EpsA-O. In the absence of EpsA-O bacteria produced weak biofilms with markedly reduced elastic and viscous moduli that correlated with a 3-log reduction in bacterial survival rate when challenged with daptomycin. These findings underscore the protective role of biofilm structure against antibiotics and suggest that targeting biofilm structural integrity could substantially enhance antimicrobial treatment strategies for biofilm-related infections.

## Introduction

Biofilms are complex, structured communities of microorganisms that adhere to surfaces and are encased in a self-produced extracellular matrix^1^. The increased tolerance to antimicrobial agents makes biofilms a major concern in both clinical and industrial settings, contributing to persistent infections such as chronic wounds and medical device-related infections^2,3^, as well as industrial issues like biofouling^4^.

The mechanisms behind biofilm antimicrobial tolerance are multifaceted, involving physical barriers from the extracellular matrix^1^, altered microenvironments within the biofilm that reduce antimicrobial penetration^5^, and the presence of dormant or slow-growing cells especially abundant in mature biofilm that are less susceptible to antibiotics^6^. Additionally, biofilms can promote the horizontal transfer of resistance genes, further complicating treatment strategies^7^. Importantly, our understanding of biofilm antimicrobial tolerance is based on the premise that bacterial cells grown in biofilm mode are significantly more resistant than planktonic bacterial cells. The two growth modes yield different bacterial community physical structures: the biofilms, where bacterial cells are closely packed and strongly mechanically interconnected and planktonic cultures, where physical distances between the individual cells are bigger and cells present much weaker mechanical connections. By measuring mechanical properties, as for example viscoelastic properties, we can probe the cohesiveness of the structure - from early mechanical coupling in the planktonic culture^8^, through the biofilm maturation process^9^, up to assessing mature biofilms^10,11^. With biofilm maturation physical structure becomes progressively more important as both elasticity and biofilm viscosity increase^9^. In addition, young biofilms consist of metabolically active bacteria that are known to be very sensitive to antibiotics^6^. Combined with low viscosity the efficacy of antimicrobials on young biofilms is relatively high. Existing research^12,13^ shows that the most resilient are mature biofilms, which are also studied in this work.

Over four decades of research^14–28^ have shown that biofilms exhibit increased tolerance to antibiotics, and many have hypothesized that the physical structure of biofilms plays a central role in this phenomenon. However, despite this long-standing assumption, the specific contribution of mature biofilm physical structure to antimicrobial tolerance has remained poorly quantified and experimentally underexplored. This is primarily due to the fact that in most existing studies, the physical structure of biofilms was not systematically manipulated or quantified. Additionally, structural factors were often confounded with physiological and environmental variables—for example, by comparing planktonic or dispersed cells to biofilm-embedded cells under different biochemical or antibiotic exposure conditions. As a result, it has remained unclear whether the structural integrity of mature biofilms alone can explain their increased antimicrobial tolerance. In fact, some studies have concluded that stationary-phase planktonic cells can be equally or even more resistant to antibiotics than biofilm cells^29^, further complicating the picture.

To directly address this gap, our study systematically investigates the role of mature biofilm structure in antimicrobial tolerance, using *Bacillus subtilis* as a model organism. The novelty of our approach lies in selectively altering the structural properties of mature biofilms while keeping biochemical and physiological conditions constant, thereby isolating the effect of biofilm structure on antibiotic tolerance. First, we performed a screening assay to identify the most efficacious antibiotic among several candidates, identifying daptomycin as the most potent. In the second part of the study, we used this antibiotic to probe how mechanical disruption and biochemical alterations of biofilm structure impact bacterial survival. Crucially, we show that the physical integrity of mature biofilms is a major determinant of antibiotic tolerance: disruption of biofilm structure alone leads to a substantial increase in daptomycin efficacy. In addition, we dissect the roles of specific biofilm matrix components, such as extracellular polysaccharide EpsA-O and the protein TasA, in shaping both the mechanical properties of the biofilm and its resistance to treatment. Together, these findings provide quantifiable evidence that biofilm physical structure—independent of other variables—is a primary contributor to the antibiotic tolerance observed in mature biofilms.

## Results

### Selection of the antimicrobial agent with the highest efficacy

**Fig. 1:**
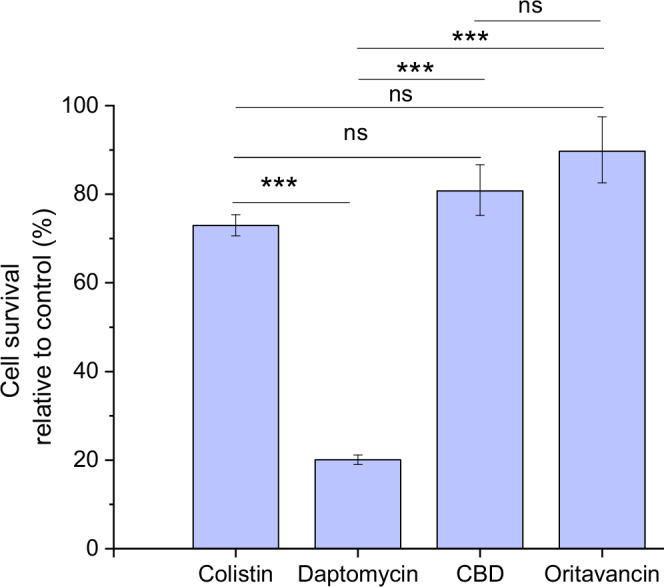
Efficacy of selected antimicrobials on native (i.e., non-disrupted) biofilms of wild-type *B. subtilis*. Based on the pre-screening test (Table 1) the high efficacy antimicrobials against fragmented biofilm suspensions were tested in native biofilms. The final antimicrobial concentration was 500 µg/mL. The results were obtained using flow cytometry; the percentage of surviving cells in the antimicrobial-treated sample was normalized to the percentage of surviving cells in the untreated controls. Daptomycin was always taken as a reference, and it was altogether tested in 29 independent biological replicates. Data are presented as mean ± standard error from at least three independent biological replicates.; ns- not statistically significant, *** extremely statistically significant.

To get a general overview of the efficacy of diverse antimicrobials aiming at different cell targets, the quick pre-screening test in microtiter plates was performed (Table 1). For this, agar- grown *B. subtilis* wild-type biofilms were fragmented using a homogenizer to obtain suspension of biofilm fragments of 1.0 - 0.1 mm in size, which preserved the biofilm microscale structure. Protein synthesis inhibitors showed low efficacy, though oxytetracycline displayed moderate efficacy. Similarly, antimicrobials targeting bacterial cell wall or DNA synthesis also exhibited low efficacy except mitomycin C and AEC-154, which had moderate efficacy. In contrast, antimicrobials affecting bacterial cell membrane integrity were most promising, except CBG (cannabigerol) and telavancin that were despite their proposed antimicrobial properties^30,31^ ineffective. The results suggest that antimicrobials targeting the cell membrane are most effective against fragmented mature *B. subtilis* biofilm suspensions. These antibiotics were therefore selected for further testing on native biofilms, where cell survival was assessed by flow cytometry. (Fig. 1). Throughout the manuscript, the native biofilms are defined as mature, intact biofilms that were not mechanically challenged prior to or during the exposure of antibiotics.

**Table 1:** Efficacy of antimicrobials on fragmented biofilm suspensions of wild-type *Bacillus subtilis*

TARGET	AB	EFFICACY
Protein synthesis	Clindamycin	-
	Linezolid	-
	Gentamicin	-
	Spectinomycin	-
	Tetracycline	-
	Erythromycin	-
	Oxytetracycline	o
Cell wall	Fosfomycin	-
	Amoxicillin	-
	Ampicillin	-
	Vancomycin	-
DNA	Mitomycin C	o
	Ciprofloxacin	-
	PPI-102-2	-
	AEC-609	-
	PPI-13-3	-
	END-85	-
	AEC-271	-
	AEC-154	o
Cell membrane	CBG	-
	Telavancin	o
	Colistin	+
	Daptomycin	+
	CBD	+
Cell wall + cell membrane	Oritavancin	+

The antimicrobials (AB) were categorized based on their cell target. Only the data at the highest antimicrobial concentration (1 mg/mL) are shown. Based on cell survival the antimicrobial efficacy (Eq. 3) was categorized into three groups: low efficacy (> 70% survival, -), moderate efficacy (40–70% survival, o), and high efficacy (< 40% survival, +). High efficacy antimicrobials were tested in 3–32 biologically independent experiments, while low and moderate efficacy antimicrobials were tested in 2–6 biologically independent experiments.

In native biofilms daptomycin showed highest efficacy (Fig. 1). Based on these results, daptomycin was selected for further experimentation.

### Impact of biofilm mechanical structure on daptomycin efficacy

**Fig. 2:**
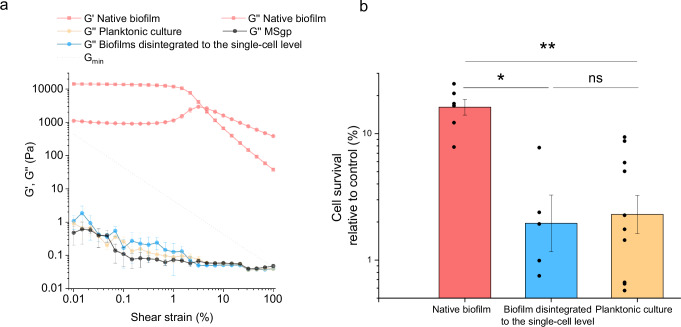
The effect of *B. subtilis* biofilm mechanical structure on daptomycin antibiotic efficacy and viscoelastic properties of biofilms. **a** The effect of mechanical structure on viscoelastic properties of wild-type (wt) *B. subtilis* biofilms; dotted line represents the boundary below which the values measured by the rheometer can become inaccurate. **b** The effect of *B. subtilis* wt biofilm mechanical structure on daptomycin antibiotic efficacy. Individual data points are shown as black dots. The antimicrobial tests on native (i.e., non-disrupted) biofilms were performed in the same set of experiments as biofilms disintegrated to the single-cell level. Data are presented as mean ± standard error from at least three independent biological replicates. ns not statistically significant, *statistically significant, **highly significant.

It is important to note that despite using extremely high concentration of daptomycin (about 100-fold MIC, Supplementary Fig. 1), the survival of cells in the native *B. subtilis* biofilms remained high (Fig. 1). To test if this is due to the biofilm physical structure, the efficacy of daptomycin was tested against native biofilms, biofilms mechanically disintegrated to the single-cell level and planktonic cultures. The experiment was designed to keep the system’s volume, the physiological state of the cells, the number of cells, the presence of exopolymers, and the antibiotic concentration constant in both native and disintegrated biofilms. What changed was the mechanical/physical state of the culture, achieved through mild sonication of the native biofilms (Fig. 2a). The mechanically disintegrated biofilms have lost all the elasticity as the storage modulus (G′) was below the detection limit. At the same time, also loss modulus (G″) substantially dropped and become comparable to the G″ of growth medium. The drop of the moduli in sonicated biofilms signifies the disruption of strong intercellular connections characteristic for structured native biofilms. The gel-like behavior of the native biofilm turned into the liquid-like behavior of biofilms disintegrated to the single-cell level, typical for bacterial suspension. The collapse of the biofilm physical structure was accompanied by the significant decrease (*p* = 0.01) in survival of the cells when exposed to daptomycin (Fig. 2b). The efficacy of daptomycin in degraded biofilm was at the comparable level to the planktonic culture. The results indicate that substantial mechanical disruption of the biofilm physical structure greatly facilitates the access of daptomycin to bacterial cells and enhances its efficacy.

### Impact of biofilm biochemical structure on daptomycin efficacy

**Fig. 3:**
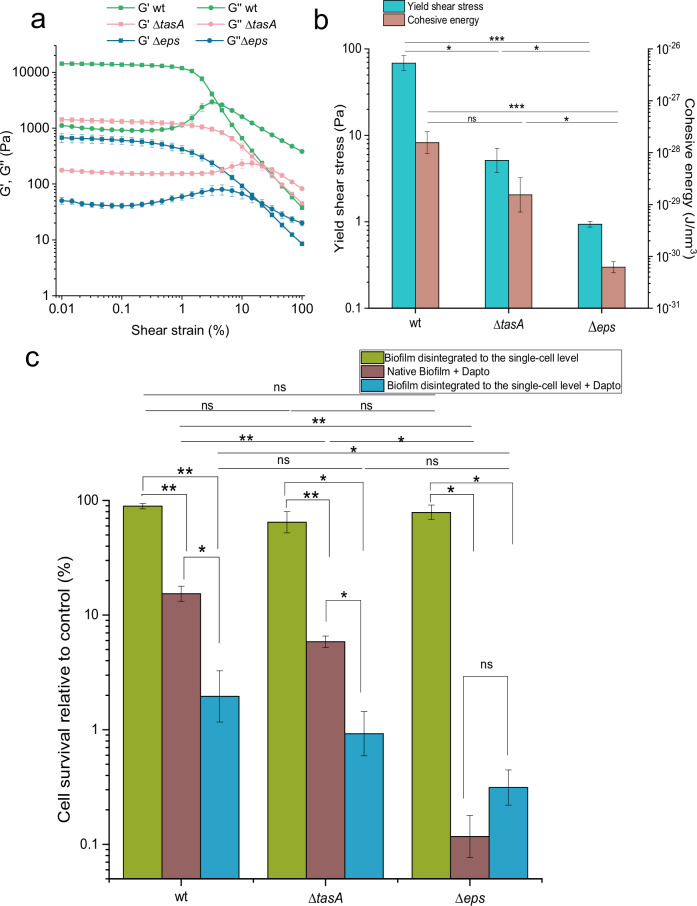
The role of biofilm biochemical structure, manipulated through application of different *B.subtilis* mutants, in mechanical properties and daptomycin efficacy. **a** The storage (G′) and loss (G″) moduli as a function of shear strain in native (i.e., non-disrupted) wild-type (wt) and mutant biofilms. **b** Yield shear stress and cohesive energy (CE) in native wt and mutant biofilms. **c** Daptomycin efficacy in native biofilms and in biofilms disintegrated to the single-cell level in wt and mutants. To see the effect of the biofilm disintegration process (sonication), the cell survival of disintegrated biofilm was normalized to the cell survival of native biofilm. To see the effect of daptomycin on native biofilm, the cell survival of daptomycin treated native biofilm was normalized to the cell survival of untreated native biofilm. To see the effect of daptomycin on biofilm disintegrated to the single cell level, the cell survival of daptomycin treated disintegrated biofilm was normalized to the cell survival of untreated disintegrated biofilm. The quantification method used was flow cytometry. Data are presented as mean ± standard error from at least three independent biological replicates; ns- not statistically significant, *statistically significant, **highly significant. *Δeps* mutant lacks the ability to produce the exopolysaccharides encoded by the *epsA-O* operon; *ΔtasA* mutant does not produce TasA, a major extracellular protein component of *Bacillus subtilis* exopolymeric substances (EPS).

In addition to mechanical disruption, we have altered biofilm structure biochemically by removing key extracellular polymeric substances (EPS), polysaccharide EpsA-O and protein TasA, involved in biofilm formation in wild-type *B. subtilis*. The native biofilms of tested mutants, deficient in EPS, exhibited substantial differences in their mechanical properties. (Fig. 3a, Supplementary Fig. 2a, b). By measuring the viscosity and viscoelasticity of native biofilms, we observed that the storage and loss moduli of the wild type (wt) were approximately one order of magnitude higher than in Δ*tasA* or Δ*eps* mutant, suggesting that the studied EPS are crucial for the structural integrity of the biofilm. The cohesive energy that represents the energy required to irreversibly deform biofilm is shown in Fig. 3b. Deleting the genes responsible for the formation of TasA or EpsA-O decreased cohesive energy of the biofilm by one or two orders of magnitude, respectively. The same was true for yield stress.

The altered mechanical properties of *B. subtilis* mutant biofilms (Fig. 3b) correlated well with their resilience to daptomycin as determined by flow cytometry (Fig. 3c) and confirmed by spread plate method (Supplementary Fig. 3). In *B. subtilis* mutant that did not produce the extracellular protein TasA (Δ*tasA*), the efficacy of daptomycin significantly improved compared to the wt (*p* = 0.003), suggesting that TasA contributes to the biofilm’s protective function against antimicrobial agents. Daptomycin was most effective against the Δ*eps* mutant that did not produce extracellular polysaccharides encoded by the *epsA-O* operon (*p* = 0.015 against Δ*tasA* and *p* = 0.005 against wt). At the same time the biofilm of this mutant appears to be the most vulnerable to mechanical stress, as it has the lowest cohesive energy and yield shear stress.

The effect of biochemical change on biofilm antimicrobial and rheological properties was determined also in biofilms mechanically disintegrated to the single-cell level (Fig. 3c, Supplementary Fig. 4a, b). As expected, mechanical disruption markedly decreased cohesiveness of the biofilms to the levels beyond the detection limit for storage modulus, G′ (Supplementary Fig. 4b). Also, the viscosity of disintegrated biofilms was strongly reduced (for about 3 orders of magnitude) to the levels where most of the biofilms approached the viscosity of solvent, MSgp (Supplementary Fig. 4a). Consistently, compared to the native biofilm the efficacy of daptomycin increased for all *B. subtilis* bacterial strains (Fig. 3c) except for the Δ*eps* mutant in which the survival rate was already very low in native biofilm, and biofilm disintegration did not change it significantly (*p* = 0.45).

At the same time the effect of disintegration process by sonication (green bars, Fig. 3c) on cell survival is negligible compared to the effect of daptomycin (brown bars, Fig. 3c). In fact, the effect of disintegration on cell survival does not significantly differ (*p* > 0.11) from 100% cell survival in any of mutants, indicating the effect of sonication on cell survival, even if present, must be small. We could not detect significant difference (*p* > 0.38) in the effect of disintegration process among the three bacterial strains. Analyzing the fraction of propidium iodide (PI) stained cells from all stained cells (PI + SYTO9) does not show the cells of disintegrated biofilms have higher fraction of PI stained cells compared to native biofilms. In general, PI stains only the cells with compromised membranes, while SYTO9 can cross intact membranes. Therefore, our results indicate the permeability of the membranes was not increased significantly (*p* > 0.45) by sonication process (Supplementary Fig. 5).

In line with the disintegration process results obtained by flow cytometry, microscopy images taken immediately after disrupting native biofilms to the single-cell level (Supplementary Fig. 6) confirm the effectiveness of sonication. The resulting bacterial suspensions contained fewer than 10% PI-stained cells and showed no visible aggregates, indicating that sonication produced a viable single-cell suspension.

**Fig. 4:**
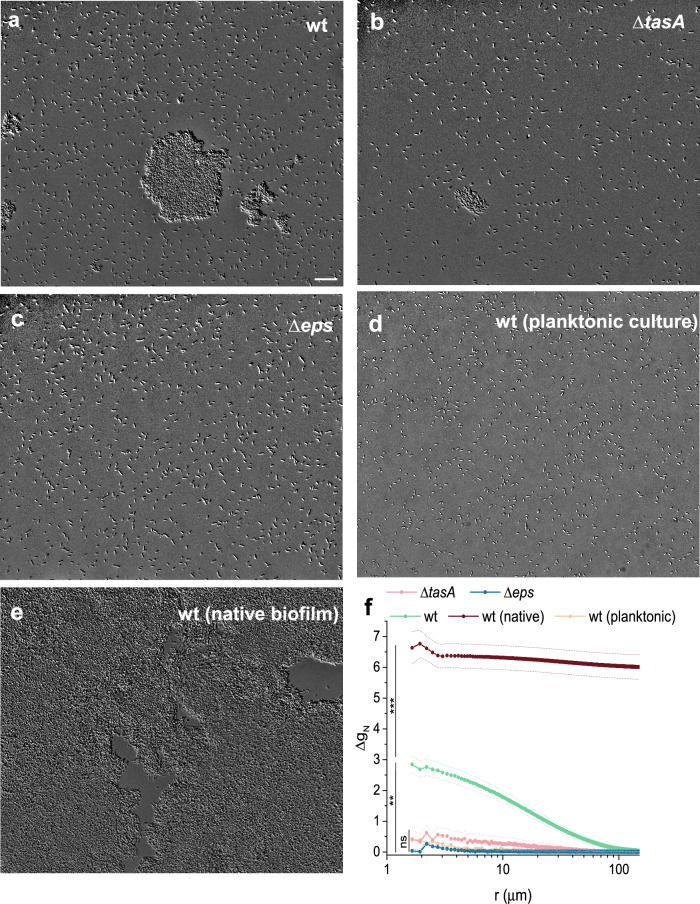
Aggregation potential of *B. subtilis* biofilm cells. **a**–**c** bacterial suspensions showing varying degrees of aggregation after the corresponding biofilms were disintegrated to the single-cell level and prepared (concentrated) for application of daptomycin, along with native biofilm and planktonic culture of the wild type in (**d**, **e**). Scale bar is the same for all images and denotes 20 μm. **f** Normalized pair-wise autocorrelation function (Δg_N_) applied to bacterial positions in microscopy images; data are presented as mean ± standard error from at least five independent samples; ns not statistically significant, **highly significant, ***extremely significant.

However, when these suspensions were subsequently prepared for daptomycin treatment, a mutant-dependent aggregation potential was observed (Fig. 4). The preparation involved a concentration step to match the volume of native agar-grown biofilms, which likely promoted aggregation in certain *B. subtilis* mutants. In both planktonic wt cultures and disintegrated biofilms of the *Δeps* mutant, bacteria remained solitary, without visible contact with neighboring cells. In contrast, aggregation was evident in the wt and *ΔtasA*, samples, where some bacterial clusters of varying shapes were observed (Fig. 4). Nevertheless, most cells in all disintegrated biofilm samples prepared for daptomycin treatment were solitary, as shown in Supplementary Table 1.

To quantify spatial correlations of bacterial cells a pair-wise autocorrelation (Eq. 9) was calculated for acquired microscopy images (Fig. 4f). There was almost no autocorrelation for Δ*eps* disintegrated biofilms, and for planktonic culture, confirming no structure was present in these samples. Albeit statistically insignificant, disintegrated Δ*tasA* biofilms indicated weak autocorrelation at small distances, in agreement with occasional presence of small bacterial aggregates (Fig. 4b). The correlation length suggests the presence of aggregates up to 40 μm in size. The largest size of the aggregates was observed in wt, where the correlation length extends to 120 μm. As we expected that the observed aggregation potential is linked to the presence of EpsA-O, we checked for the possible presence of this polysaccharide by staining the sample by Indian ink dye^32^. The micrographs confirmed our expectations (Supplementary Fig. 7): the cells of disintegrated biofilms of wt, Δ*tasA* and planktonic culture contained some extracellularly cell attached material, likely EpsA-O, which was, however, not present in daptomycin most sensitive Δ*eps* mutant.

Our results suggest that biochemical disruption of biofilms by removing key extracellular polymers considerably alters their mechanical properties and tolerance to daptomycin. Mutants lacking TasA or EpsA-O showed decreased cohesive energy and yield shear stress, making them more susceptible to daptomycin. The *Δeps* mutant, which lacks extracellular polysaccharides, was the most vulnerable to both mechanical stress and daptomycin, highlighting the protective role of these polymers. Additionally, biofilms disintegrated to the single-cell level, exhibited reduced viscosity and cohesiveness, further enhancing daptomycin efficacy.

### Impact of biochemical structure on the dynamics of daptomycin efficacy in native biofilms

**Fig. 5:**
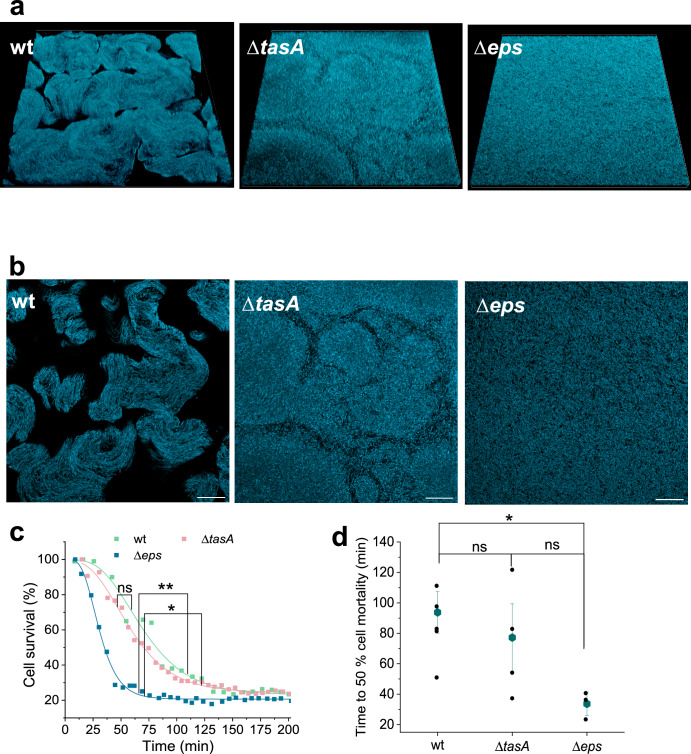
The effect of extracellular biochemical structure on biofilm topology and dynamics of daptomycin efficacy. **a** 3D-reconstructed CLSM maximum intensity projection images of 150 μm × 150 μm × 12 μm of upper layers of native *B. subtilis* biofilms. **b** Optical CLSM slices through native *B. subtilis* biofilms. Scale bar represents 20 μm. **c** Dynamics of cell survival after daptomycin treatment over time of *B. subtilis* native biofilms. Curves represents fitted curves of average cell survival at specific time point. **d** Time to 50% cell mortality of native *B. subtilis* biofilms; individual biological measurements are shown as black dots together with mean ± standard error. ns not statistically significant, *statistically significant, **highly significant.

To check how daptomycin antimicrobial dynamics is affected by impaired extracellular biochemical structure we have grown native biofilms and monitored cell survival inside the biofilm with time-lapse CLSM (Confocal laser scanning microscopy) imaging (Fig. 5). Different morphologies of native biofilms are shown in Fig. 5a, b. One can observe 3D wrinkled surface structure in wt that is completely absent in Δ*eps* mutant, which had flat homogenous biofilm surface (Fig. 5a). The optical cut through the biofilm physical structure reveals that bacterial strains with intact *eps* formed locally condensed structures that were absent in Δ*eps* mutant, suggesting that polysaccharide EpsA-O is essential for the formation of locally tightly packed cell clusters. At the same time mutants lacking *tasA* cannot form as tightly packed structures as in the case of wt, suggesting that protein TasA is also involved in formation of locally tightly packed cell clusters. The antimicrobial action of daptomycin was most rapid on the Δ*eps* mutant (Fig. 5c, d). After addition of daptomycin cell survival rapidly decreased, and most of the cells in the biofilm were dead after ~60 min of the treatment (Supplementary Fig. 8). In contrast, the wild type and Δ*tasA* mutant, displayed a much slower rate of cell dying, requiring over 140 min for daptomycin to be fully effective (Fig. 5c, Supplementary Fig. 8). Similar dynamics was observed for the wt native biofilms. These results further strengthen the crucial role of polysaccharide EpsA-O in protecting biofilms against antimicrobial agents.

## Discussion

The findings from this study provide a critical link between biofilm structure and the efficacy of antimicrobial agents. Although biofilms in tissues or on medical devices are exposed to more complex fluid dynamics and host factors, our simplified and controlled system allows us to isolate the effects of matrix structure and biofilm organization on antimicrobial tolerance. Importantly, our results—such as the protective role of biofilm architecture—are consistent with mechanisms proposed in clinically relevant settings and may inform future studies in more complex models. In particular, our data enabled quantitative assessment of the contribution of biofilm physical structure to antimicrobial tolerance.

The microtiter plate pre-screening of various antibiotics on fragmented *B. subtilis* biofilm suspensions revealed that the antibiotics targeting metabolically active components were ineffective, even though most of them were added in concentrations 100–1000 times of MIC. This is not surprising, as it is known that non- or slow-growing bacteria are less sensitive to antimicrobials^33,34^. It is believed that limited penetration of nutrients rather than restricted access for antibiotics contribute to the general antimicrobial tolerance observed in biofilms^25^. On the other hand, targeting the bacterial membrane that is essential for bacterial survival regardless of the growth state appeared more promising in fragmented biofilms tests. The finding that colistin was among the most effective antibiotics when attacking fragmented biofilm suspensions was unexpected, as it is classically known to target Gram-negative bacteria by interacting with lipopolysaccharides, which are absent in Gram-positive bacteria like *B. subtilis*. On the other hand, some recent studies have reported activity of colistin against certain Gram-positive bacteria. Notably, *Paenibacillus polymyxa*, a close relative of *B. subtilis*, has been shown to be susceptible to colistin through a mechanism involving membrane disruption^35^. Furthermore, a study by the same group suggested that colistin-induced killing of *B. subtilis* involves enhanced NADH metabolism and membrane damage^36^. However, when the intact mature biofilms were exposed to the membrane attacking antibiotics, only daptomycin was able to kill the majority of the biofilm cells, consistent with its current status as an antibiotic of choice for the treatment of biofilm-related infections in gram-positive pathogenes^37–41^. Still, a substantial portion ( ~ 20%) of biofilm cells survived the daptomycin treatment, suggesting an additional tolerance mechanism that complements low metabolic activity of bacterial cells in the biofilm and contributes considerably to increased antimicrobial tolerance in biofilms. After the native wt biofilm was mechanically disintegrated to the single-cell level and cells were exposed to daptomycin under the same conditions as in the native biofilms, only 2% of persisters were recovered, suggesting that the biofilm physical structure is the main tolerance mechanism in tested biofilms. Consistently, mutant biofilms, deficient in one of the key exopolymeric substances (EPS), were less resistant to shear stress, had lower cohesive energy, showed less structural features, and were at the same time substantially less tolerant to daptomycin. These results are in line with recent study where Nahum et al.^42^ linked variability in *Pseudomonas aeruginosa* biofilm antimicrobial tolerance to changes in biofilm mechanical properties. The viability of less stiff biofilms was largely unaffected by tobramycin. However, when tobramycin was combined with low-frequency ultrasound, the inactivation of the biofilms increased substantially. For stiffer biofilms higher low-frequency ultrasound intensities were needed to achieve enhanced antibiotic susceptibility.

Our results show that biofilm antimicrobial tolerance is related to the type of EPS present in the biofilm. Biofilms lacking TasA protein, which contributes to the biofilm formation^9,43,44^, were, compared to wt biofilms mechanically weaker, displayed less pronounced 3D structural features and were much more sensitive to daptomycin. Although MIC in Fig. S2 indicate that mutations do not alter the basic susceptibility to daptomycin in mutants, the contribution of non-mechanical causes to decreased antibiotic tolerance in the biofilm cannot be fully excluded, as recent research shows that in *tasA* mutant expression of several stress genes is altered^45^. On the other hand, biofilms lacking exopolysaccharide EpsA-O (Δ*eps*) were mechanically the weakest, displayed no 3D structural features and were the most sensitive to daptomycin. Compared to biofilms lacking TasA, the biofilms lacking polysaccharide EpsA-O were significantly more sensitive to daptomycin (*p* = 0.015). Therefore, our results strongly indicate that exopolysaccharides, rather than proteins, are the primary extracellular polymers responsible for the biofilm’s protective barrier against antimicrobials. The critical role of extracellular polysaccharides in antimicrobial protection has also been demonstrated in *Pseudomonas aeruginosa*, where these components substantially contribute to biofilm-associated antimicrobial tolerance^46,47^.

In conjunction with the recently discovered unique primary structure of the polysaccharide EpsA-O^32^, one might be tempted to conclude that the mere presence of EpsA-O is sufficient to decrease the efficacy of daptomycin in biofilms. A possible mechanism could be binding of daptomycin with its positive charged aliphatic primary amine^48^ to negatively charged pyruvate decorated sugar residues in the side chains of EpsA-O^32^. However, the mere presence of EpsA-O appears to be insufficient to fully account for a decrease in the efficacy of daptomycin. The results indicate that although samples of native biofilms and biofilms mechanically disintegrated to the single-cell level have the same amount of EpsA-O, the disintegrated biofilms are substantially more sensitive to daptomycin. It appears that EpsA-O has a double role in antimicrobial tolerance. It provides a structural support and reduces diffusion of the antibiotic in the biofilm. When biofilm structure is disintegrated EpsA-O still provides almost an order of magnitude more tolerance to daptomycin compared to cells that lack EpsA-O (*p* = 0.036). In all disintegrated biofilms except in Δ*eps* biofilms, extracellular material attached to the cell surface was observed, strongly suggesting that at least some of EpsA-O remained cell bound. All of these suggest that once the biofilm physical structure has collapsed the EpsA-O can still offer some antimicrobial protection to the cells. However, the protection provided by EpsA-O attached to a single cell is minimal compared to the protective effect of the biofilm’s physical structure. Therefore, the primary role of exopolysaccharide EpsA-O in biofilm antimicrobial protection is through building of biofilm physical structure.

One possible mechanism by which the physical integrity of *B. subtilis* biofilms enhances their resilience is through the action of the hydrophobic protein BslA. BslA forms a hydrophobic coat on the biofilm surface^49^, which could impede the penetration of aqueous antimicrobials such as daptomycin. Upon biofilm disintegration, this surface layer is likely disrupted, potentially facilitating increased antibiotic penetration.

Although no studies have demonstrated a direct physical interaction between the EpsA–O polysaccharide and BslA, several lines of evidence suggest functional interdependence. For example, mechanical coupling between matrix components has been proposed^50,51^, and altered expression of *bslA* has been observed in *epsA–O* mutants^52,53^. These findings point to the importance of EpsA–O not only for structural integrity but also for the assembly or maintenance of the BslA-mediated barrier, thereby contributing to overall biofilm resilience.

An important question in bacterial physiology and antibiotic therapy is the possible presence of VBNC cells in biofilms. Although our experiments were not primarily designed to identify VBNCs, comparing results from flow cytometry and the spread plate method provides valuable insight. Qualitatively, both methods gave similar trends (Fig. 3c vs. Supplementary Fig. 3), but colony-forming unit (CFU)-based counts were consistently lower, suggesting the presence of VBNC cells. This discrepancy was most prominent in disintegrated wt biofilms, where the CFU drop exceeded that observed by flow cytometry. This may indicate either that biofilm structural loss promotes a transition into the VBNC state, or that viable cells depend more strongly on the biofilm structure for protection than VBNC cells do, making them more vulnerable to daptomycin once the biofilm is disrupted.

In conclusion, our results support the hypothesis that in mature *B. subtilis* biofilms, the key factor enabling microbial tolerance to antibiotics is biofilm physical structure. The absence of biofilm structural integrity lowers the bacterial survival rate, which can be further lowered by removing exopolysaccharide EpsA-O. Our findings suggest that in order to fight the mature biofilms successfully the treatment should include a combination of weakening of the physical structure of the biofilm followed by antibiotics that target cell structures that are vital both for metabolically active and inactive cells in the mature biofilms such as membrane targeting daptomycin.

## Methods

### Bacterial strains and growth conditions

All bacterial strains used in this study are listed in Table 2. The BFP-labeled *Bacillus subtilis* NCIB 3610 strains were constructed by transformation of competent derivative NCIB 3610 *comI* ^*Q12L*^^54^ with plasmid pNW2304 carrying *mTag-BFP* fluorescent protein expressed from the constitutive P_hyperspank_ promoter flanked by the *amyE* locus^55^, kindly provided by N. Stanely-Wall. *mTag-BFP* fluorescent protein with spectinomycin resistance cassette was integrated into the NCIB 3610 *comI* ^*Q12L*^ genome. Mutations were then introduced into BFP-labeled strain by transformation with DNA derived from NCIB 3610 strains carrying Δ*epsA-O*::*tet*; Δ*tasA* labeled derivative was obtained by transformation with PCR product amplified from chromosomal DNA isolated from the bacterial strain BKE24620 carrying Δ*tasA*::*erm* construct by using primers tasA_5pL (5′ to 3′: GATACGAAAGGCACTGTATGCTC) and tasA_3pR (5′ to 3′: GACTATTGCTTCACCATTTCACC)^56^. Appropriate antibiotics were used for bacterial strain constructions and experiments at the following concentrations: 10 μg/mL of tetracycline, 100 μg/mL of spectinomycin, 10 μg/mL of kanamycin, and 10 μg/mL of erythromycin.

**Table 2:** Bacterial strains used in this study

Strain	Genotype^a^	Source/construction^b^
NCIB 3610	*B. subtilis* wild type - prototroph	*Bacillus* Genetic Stock Center
DL4	*B. subtilis* NCIB 3610 *(epsA-O)::tet*	^43^
DL963	*B. subtilis* NCIB 3610 *tasA::spec*	^43^
DK 1042	*B. subtilis* NCIB 3610 *comI* ^*Q12L*^	^54^
BM2011	*B. subtilis* NCIB 3610 *comI* ^Q12L^ *amyE::Phyperspank- mTagBFP (spec)*	*this work* pNW2304 → DK 1042
BM2012	*B. subtilis* NCIB 3610 *comI* ^Q12L^ *amyE::Phyperspank- mTagBFP (spec) (epsA-O)::tet*	*this work* DL4 → BM2011
BM2013	*B. subtilis* NCIB 3610 *comI* ^Q12L^ *amyE::Phyperspank- mTagBFP (spec) ΔtasA::erm*	*this work* BKE24620 → BM2011
BKE24620	*B. subtilis* 168 *trpC2*Δ*tasA::erm*	^56^
NRS6930	*E. coli DH5α* pNW2304 *(amyE:: Phyperspank -mTagBFP (spec)* inserted in pDR111 *(amp)*	^55^

^a^Drug resistance cassette: *tet* tetracycline resistance, *spec* spectinomycin resistance, *kan* kanamycin resistance, *erm* erythromycin resistance, *amp* ampicillin resistance

^b^Strain construction is indicated as a source of DNA (donor strain or plasmid) transformed into recipient strain (indicated by the arrow). BKE strain was obtained from the BKE library^56^.

Overnight cultures were grown in LB liquid medium (tryptone 10 g/L; yeast extract 5 g/L; NaCl 5 g/L) without antibiotics at 28 °C with shaking (200 rpm) for 24 h.

To obtain native biofilms, bacterial cultures were grown either on solid MSgg agar plates or in custom 3D-printed 8-well chambered slides^32^ filled with MSgg agar (100 mM MOPS (3-(N-morpholino) propane sulfonic acid); 5 mM K_3_PO4; 50 mg/L tryptophan; 50 mg/L phenylalanine; 2 mM MgCl_2_ ∙ 6H_2_O; 0.5% (w/v) sodium glutamate; 0.5% (w/v) glycerol; 700 μM CaCl_2_ ∙ H_2_O; 50 μM FeCl_3_ ∙ 6H_2_O; 50 μM MnCl_2_; 1 μM ZnCl_2_; 1.5% agar; 2 μM thiamine hydrochloride. The pH was adjusted to 7.0. All components of the medium, except thiamine hydrochloride, were autoclaved together at 110 °C). Custom 3D-printed chambered slides^32^ (25.5 mm × 72.5 mm) were made of PET-G. Each slide contained 8-well chambers (9.6 mm × 10.5 mm) with depth of 3.3 mm.

Petri dishes (90 mm diameter) were filled with 20 mL of MSgg agar, while each well in the 3D-printed slide contained 300 µL of the agar. Overnight cultures were diluted 1:64 in MSgp medium. MSgp is a modified MSgg medium that does not contain any nitrogen source, i.e., no amino acids, glutamate is replaced by equal amount of sodium pyruvate. This substitution limits uncontrolled bacterial growth while largely preserving the physico-chemical properties of the medium, such as pH and osmolarity. 250 µL of the diluted overnight culture was transferred onto the MSgg agar plates, while a 4 µL inoculum was added to each agar-filled well. To ensure uniform biofilm formation, the inoculum was spread using glass beads (one bead per well for the chambered slides), followed by incubation in a climatic chamber (ICH260L, Memmert, Germany) at 37 °C and 80% relative humidity for (15 ± 1) h. After this period, the biofilm mass did not increase, indicating it had reached the mature stage.

For planktonic culture samples, overnight cultures were diluted 1:64 in MSgp medium. A volume of 250 µL of diluted culture was then added to 20 mL of fresh MSgg and incubated at 37 °C with shaking at 200 rpm for (15 ± 1) h.

### Selection of the antimicrobial agent with the highest efficacy

The aim of this pre-screening microtiter plate assay was to select for the most efficacious antimicrobial agent against *Bacillus subtilis* biofilm. For this purpose, biofilms of *B. subtilis* wild type (wt) were first grown on MSgg agar plates and then scraped off the agar surface using a glass microscope slide. After determining their mass on an analytical scale, the collected biofilms were diluted in MSgp medium at a 1:56 (w/v) ratio, followed by homogenization process. This was achieved by processing the biofilm suspension with an Ultra-Turrax T8 homogenizer at level 4 for 20 s. In this way we obtained homogenized biofilm suspension consisting of biofilm fragments of 1.0–0.1 mm in size, which preserved the biofilm microscale structure and was suitable for microtiter plate assay. For positive control samples, 1 mL of the homogenized biofilm suspension was transferred into Eppendorf tubes and heated in a dry thermoblock at 77 °C for 30 min. Untreated biofilm, unexposed to antimicrobials, served as the negative control.

Exposure to the antimicrobial agents was conducted in 96-well microtiter plates. Each well contained 100 μL of fragmented biofilm solution, 50 μL of the antimicrobial agent, 40 μL of propidium iodide (PI: 0.8 mM), and 60 μL of MSgp. Antimicrobials were tested at final concentrations of 1 mg/mL, 0.5 mg/mL, and 0.1 mg/mL.

Antimicrobials were prepared as 5 mg/mL stock solutions in MSgp medium. To dissolve antimicrobials insoluble in pure MSgp, DMSO was added (Supplementary Table 2). PPI-102-2, AEC-609, PPI-13-3, END-85, AEC-271, AEC-154^57^ were kindly provided by prof. dr. Nace Zidar; CBG (cannabigerol) and CBD (cannabidiol) were kindly provided by ICANNA Institute, Slovenia; the rest of antibiotics were commercially available (Supplementary Table 2).

Fluorescence measurements were taken over a 17-h period at 15-min intervals (for a total of 69 readings) using the BioTek Cytation 5 Cell Imaging Multimode Reader. Plates were shaken at 425 rpm prior each reading. To follow the fluorescence of PI, the excitation was set at 535/20 nm, and emission was recorded at 617/20 nm from both the top and bottom of the wells, with gain settings of 90 and 110, respectively. Additionally, optical density (OD) was measured at *λ* = 650 nm.

When analyzing the recorded fluorescence, we took into account the background and normalized the fluorescence per unit OD.

The fluorescence intensity at each time point for sample X (either antimicrobial-treated, S, or untreated control cells, C_n,_ both with added PI) was normalized using the following equation:

1 $${{\rm{F}}}_{\mathrm{norm}({\rm{X}}+\mathrm{PI})}=\frac{{{\rm{F}}}_{({\rm{X}}+\mathrm{PI})}-{{\rm{F}}}_{\mathrm{avg}\,\left(\mathrm{MSgp}+\mathrm{PI}\right)}-{{\rm{F}}}_{\mathrm{avg}\,({{\rm{c}}}_{{\rm{p}}})}\,}{{\mathrm{OD}}_{({\rm{X}}+\mathrm{PI})}-{\mathrm{OD}}_{\mathrm{avg}\,(\mathrm{MSgp}+\mathrm{PI})}\,}$$

F denotes fluorescence intensity as measured by spectrofluorometer, Cp denotes positive control sample (treated cells at 77 °C), PI, added propidium iodide, OD optical density. OD_(X+PI)_ is optical density corresponding to the wells where F_(X+PI)_ was measured. For all time points the value of OD_(X+PI)_ at the start of the experiment (*t* = 0 h) was taken into account. This is necessary, as these samples contained cells that could decay with time, decreasing OD and thus falsely indicate lower biomass in the microtiter well. MSgp denotes the samples without cells i.e., only with MSgp medium; index avg designates the average over at least 3 wells of a microtiter plate.

The fluorescence intensity for the positive control samples with PI, (Cp + PI), was normalized applying the following equation:

2 $${{\rm{F}}}_{\mathrm{norm}({{\rm{C}}}_{{\rm{p}}}+\mathrm{PI})}=\frac{{{\rm{F}}}_{({{\rm{C}}}_{{\rm{p}}}+\mathrm{PI})}-{{\rm{F}}}_{\mathrm{avg}\left(\mathrm{MSgp}+\mathrm{PI}\right)}-{{\rm{F}}}_{\mathrm{avg}({{\rm{C}}}_{{\rm{p}}})}\,}{{\mathrm{OD}}_{({{\rm{C}}}_{{\rm{p}}}+\mathrm{PI})}-{\mathrm{OD}}_{\mathrm{avg}(\mathrm{MSgp}+\mathrm{PI})}\,}\cdot \,\frac{{\mathrm{OD}}_{\mathrm{avg}({{\rm{C}}}_{{\rm{p}}}+\mathrm{PI})}-{\mathrm{OD}}_{\mathrm{avg}(\mathrm{MSgp}+\mathrm{PI})}\,}{{\mathrm{OD}}_{\mathrm{avg}({{\rm{C}}}_{{\rm{n}}}+\mathrm{PI})}-{\mathrm{OD}}_{\mathrm{avg}(\mathrm{MSgp}+\mathrm{PI})}\,}$$

The heat applied to the positive control samples can induce different biomass to OD ratio. Therefore, OD of heat-treated positive control sample was corrected by the second factor in the Eq. 2, which normalizes OD of heat-treated positive control sample with OD of negative control. Note that both samples contained the same amount of biomass. OD_avg (Cn+PI)_, OD_(Cp+PI)_ and OD_avg (Cp+PI)_ refer to the measurements at *t* = 0 h.

Finally, the cell survival was calculated using the previously obtained average normalized values (Eq. 1 and Eq. 2):

3 $$\mathrm{Cell\; survival}\,\left( \% \right)=100 \% \cdot(1-\frac{{{\rm{F}}}_{\mathrm{norm}({\rm{S+PI}})}-{{\rm{F}}}_{\mathrm{norm}({{\rm{C}}}_{{\rm{n}}}+\mathrm{PI})}\,}{{{\rm{F}}}_{\mathrm{norm}({{\rm{C}}}_{{\rm{p}}}+\mathrm{PI})}\,})$$

For the final presentation of the pre-screening results, we categorized antimicrobial efficacy based on cell survival (Eq. 3) into three groups: low efficacy (survival > 70%), moderate efficacy (survival 40–70%), and high efficacy (survival < 40%). Only antimicrobials in the last category were considered potentially effective against native biofilms.

### Evaluating antibiotic efficacy in native biofilms, biofilms mechanically disintegrated to the single-cell level and planktonic cultures

For native (i.e., non-disrupted) biofilms, grown in 8-well chambered slides, 30 µL of a 5 mg/mL antimicrobial agent was applied after a (15 ± 1) h incubation period. Control biofilms on the same slide received 30 µL of MSgp. The slides were placed in empty Petri dishes, sealed with parafilm to prevent evaporation, and incubated at room temperature for 4 h. After incubation, biofilms were collected from the agar using a custom 3D-printed plastic strip or a scalpel blade and transferred to 1 mL of MSgp medium (yielding a final concentration of ~5 mg/mL or 10^9^ cells/mg of biofilm).

For the studies where we investigated the impact of biofilm physical structure on antimicrobial resilience we used only daptomcyin. For biofilms that were mechanically disintegrated to the single-cell level, native biofilms were obtained by incubation on MSgg agars in parallel to MSgg liquid culture that served to obtain MSgg spent medium. The spent medium was prepared by adding 15 mL of fresh MSgg to 200 µL of a 64x diluted overnight culture in a 70 mm Petri dish, incubated at 37 °C with 80% humidity alongside the biofilms. After incubation, the native biofilms were transferred into 1 mL of spent and filtered (0.2 µm) MSgg medium and sonicated for 10 s at an amplitude of 12 microns by 150 Watt Ultrasonic Disintegrator Mk2 (MSE Scientific Instruments) with 3 mm exponential probe. The used sonication protocol was optimized on native wt *B. subtilis* biofilms to give cell suspensions with high survival (> 90%) and devoid of aggregates (see Testing the sonication protocol used for biofilm disintegration). These samples were then centrifuged at 8000 × *g* for 5 min, after which 700 µL of the supernatant was discarded. The remaining suspension was vortex-stirred and 30 µL of a 5 mg/mL daptomycin was added, followed by incubation at room temperature for 4 h. The disintegrated biofilm cells were during this time in MSgg spent medium to mimic spent MSgg agar for native biofilm comparison. After incubation, the samples were centrifuged again at 8000 × *g* for 5 min, and the spent medium was replaced with 1 mL of MSgp.

Prior to flow cytometry, both native biofilms and disintegrated biofilms were sonicated for 10 s at an amplitude of 12 microns in order to obtain single cell suspensions, suitable for flow cytometry analysis. The samples were then diluted 20-fold with MSgp and stained by Live/Dead staining kit (stock solutions of PI and SYTO9, diluted 5000-fold; Thermo Fisher Scientific) to subsequently assess cell viability using flow cytometry. Alternatively, cell survivability was analyzed using the spread plate method. Serially diluted cultures in MSgp were spread on MSgg agar plates, which were incubated overnight at 37 °C. CFUs were counted the following day.

To assess antibiotic efficacy in planktonic cultures, 1 mL of the grown culture was transferred into Eppendorf tubes, with a minimum of three replicates for each condition. The cultures were sonicated for 10 s at an amplitude of 12 microns. After sonication, 700 µL of the culture was removed, and 30 µL of a 5 mg/mL antimicrobial agent was added to the remaining 300 µL. This volume corresponded to the volume of agar in the native biofilm experiments. The mixture was incubated at room temperature for 4 h. Following incubation, the samples were diluted 20-fold with MSgp, and cells were stained by Live/Dead staining kit, with stock solutions diluted 5000-fold to subsequently assess cell viability using a Flow cytometry.

Simultaneously, control samples were prepared. For the unstained control, filtered MSgp was mixed with an unexposed sample without the addition of the Live/Dead staining kit. For the media control, MSgp was used without any additional components. As a positive control, filtered MSgp was combined with dead biofilm or planktonic cells (prepared by heating 1 mL of biofilm or planktonic culture at 77 °C for 30 min), followed by staining with the Live/Dead kit in the same ratio as used in experimental samples.

### Testing the sonication protocol used for biofilm disintegration

The sonication protocol used to disintegrate native biofilms to the single cell level was checked on native biofilms. For this, native biofilms were sonicated (protocol is given in Evaluating antibiotic efficacy native biofilms, biofilms mechanically disintegrated to the single-cell level and planktonic culture), stained by Live/Dead staining kit (stock solutions of PI and SYTO9, diluted 5000-fold) and immediately taken for microscopy (see Visualization of mechanical structure of *B. subtilis* biofilms and planktonic culture) or were analyzed by flow cytometry (see Flow cytometry). For controls, non-disintegrated samples were analyzed.

### Flow cytometry

The samples were analyzed using the BD FACSMelody™ Cell Sorter, operated via BD FACSChorus, with 100,000 events recorded per sample. The flow rate was kept constant at 3. Prior to sorting, each sample was briefly vortexed. The sorter was configured to detect SYTO 9 and Propidium Iodide (PI) fluorochromes. The threshold for Forward Scatter Height (FSC-H) was set to 14,100.

The analysis began with the control samples: the media control, followed by the unstained control, and finally the positive control. Subsequently, all experimental samples were measured and recorded. Data analysis was performed using FlowJo_V10: Flow Cytometry Analysis Software. The first step involved distinguishing the cell population from the noise (i.e., MSgp) by setting the gate to 1000 at SSC-A. Based on control samples (medium, unstained sample, and positive control), the live cell population was gated for each sample. Only the cells stained by SYTO 9 and not PI were considered alive. Results were normalized against the controls (no antibiotic exposure) using Eq. 4, to account only for cell death induced by the tested antibiotic:

4 $${D}_{{survival}}\left( \%\right)=\frac{{{\rm{S}}}_{{\rm{A}}}}{{{\rm{C}}}_{{\rm{A}}}}\cdot 100 \%$$

where S_A_ represents the event rate of cells that survived in the biofilm following antimicrobial treatment, and C_A_ denotes the event rate of cells that survived in the control sample. The validity of flow cytometry gating approach and Eq. 4 was confirmed by simultaneously conducting spread plate count method for six native biofilm samples and six samples of biofilms disintegrated to the single cell level (*R*^2^ = 0.91). See Supplementary Fig. 9 for gating strategy. The linearity of cell event rate against cell concentration was verified by dilution method (*R*^2^ > 0.99).

### Determination of minimum inhibitory concentration (MIC)

The minimum inhibitory concentration (MIC) is the lowest concentration of a compound required to inhibit bacterial growth. After overnight incubation, the bacterial culture was diluted in LB medium to an optical density OD_650_ of ~1.0 AU. A 2.5% inoculum was transferred to fresh MSgg medium and incubated at 28 °C until the culture reached an OD_650_ of ~0.1 AU. Subsequently, 90 µL of the cell suspension was added to each well of a clear 96-well plate, followed by 90 µL of daptomycin at 1.5- to 2-fold serial dilutions, ranging from 0.0 to 75.0 µg/mL. The plates were incubated at 28 °C with shaking at 1000 × *g* for 18 h. Bacterial growth was monitored by measuring OD_650_ using a Thermo Scientific Multiskan Spectrum plate reader. The lowest concentration of daptomycin (MIC) that completely inhibited bacterial growth was determined.

### Rheology

To determine the viscoelasticity, two dynamic moduli were measured with modular oscillating rheometer Anton Paar Physica MCR 302 with a plate-plate (PP25, *d* = 25 mm) measuring system. The storage modulus (G′) measures the elastic response of the material, which measures stored energy, while the loss modulus (G″) measures the viscous response of the material, where energy is dissipated as heat. Native biofilm samples were scraped from MSgg agar plate with microscope object slide and placed on measuring surface, while for biofilms disintegrated to the single-cell level 100 μL of suspension was placed on measuring surface. The tested gap height was 0.2 mm, the measurements temperature was maintained at 20.0 °C by thermoelectric device (Peltier system, Anton Paar), and normal force was set to *F*_N_ = 2 N. Custom enclosure for measuring system was 3D printed to minimize evaporation. Amplitude sweep experiments were performed at a constant frequency of 10 rad/s and by increasing the strain amplitude from 0.01 to 100% to determine the linear viscoelastic (LVE) range. Frequency sweep experiments were measured from 0.1 to 100 rad/s at constant strain amplitude of 1% for native biofilms and at 10% strain amplitude for biofilms disintegrated to the single-cell level. 25 logarithmically spaced measuring points were captured for amplitude sweep experiments and 16 logarithmically spaced measuring points were captured for frequencies sweep experiments.

Through amplitude sweep measurements, we can determine the critical shear strain (γ_c_), which represents the maximum shear strain within the LVE region. We determined the yield stress as a stress occurring at γ_c_ and interpreted it as a pressure required to irreversibly deform the structure. In this context, G′ denotes the storage modulus at the γ_c_. Cohesive energy, which refers to the energy needed to irreversibly deform the structure was determined using following equation^32,58^:

5 $${CE}=\frac{1}{2}{G}^{{\prime} }{{\gamma }_{c}}^{2}$$

### Visualization of mechanical structure of *B. subtilis* biofilms and planktonic culture

Wild-type biofilm solutions stained by Live/Dead staining kit and obtained immediately after sonication were observed using N-Achroplan 63×/0.85 M27 objective using fluoresce contrast technique and Axiocam 705 microscopy camera installed on microscope Axio Observer 7 (ZEISS, Germany). The light source used was X-Cite XYLIS LED Illumination System, with deployed GFP Ultra Bandpass and mKate—TxRed Ultra Bandpass Filter Sets. Three independent biofilm samples were observed, by capturing mosaic images (9 tiles) corresponding to the field of view size of 600 μm × 500 μm with at least 1000 bacteria.

Native/disintegrated biofilm solutions before application of daptomycin were observed using LD Plan-Neofluar 40×/0.6 Korr M27 oil objective using differential interference contrast technique (DIC). Mosaic images (9 tiles) corresponding to the size of 945 μm × 788 μm were acquired. At least 1000 bacteria were recorded each time. For each sample, at least 5 separated mosaic images were recorded. The counting of bacteria was done by a custom script written in ImageJ macro language.

For Indian ink-stained *B. subtilis* biofilms visualization, 2 µL of native/disintegrated biofilm solutions were mixed with an equivalent volume of Indian ink (Royal Talens, Netherlands) on a microscope slide. A cover glass was placed on the slide glass and any excess fluid was pushed out using thumb pressure. The negatively stained samples were observed by using differential interference contrast technique (DIC) by Plan-Apochromat 100×/1.40 Oil DIC M27 oil objective. At least five independent experiments were performed.

### Pair-wise correlation analysis of microscopic images

The pair-wise correlation was performed similarly to the method described in ref. ^59^. The positions of individual bacteria on microscopy images were extracted by a custom written script in ImageJ. The positional coordinates served as input parameters to a C^++^ program routine dedicated to calculating the spatial autocorrelation function, which calculates the normalized pair-wise correlation function defined as:

6 $${\rm{g}}\left({\rm{r}}\right)=\frac{{\rm{\langle }}I({\rm{x}})I({\rm{y}}){\rm{\rangle }}}{{\rm{\langle }}{I}^{2}{\rm{\rangle }}}$$

where *< I(x)I(y) >* is the averaged product of fluorescence intensities of two pixels at a distance *r* and < *I*^2^ > is the averaged square of intensities of all pixels, making *g*(0) = 1. The function returns the probability of finding two bacteria at a distance *r* on the analyzed microscopy image. For a completely random position of bacteria (i.e., the density of bacteria is the same on all scales and directions), one can expect that the probability of finding two bacteria at certain distance will equal to the ratio of bacteria (non-background pixels) to the image size (all pixels):

7 $${{\rm{g}}}_{{rand}}(r)=\frac{{{\rm{N}}}_{{\rm{bacteria}}}}{{{\rm{N}}}_{{\rm{tot}}.{\rm{pixels}}}}$$

The function is valid for *r* > 1 pixel; at *r* = 0, by definition the function returns 1. To correct for the random distribution of bacteria, we have subtracted *g*_*rand*_*(r)* from *g(r)*:

8 $$\Delta {\rm{g}}({\rm{r}})={\rm{g}}({\rm{r}})-{{\rm{g}}}_{{rand}}({\rm{r}})$$

The positive values of *∆g*(r) indicate a non-random distribution of bacteria. The probability to find a pair of bacteria at a certain distance is not only a function of distribution of bacteria, but also a function of a bacterial density. The more bacteria that are in the image, the more likely it is to encounter a bacterial pair at a certain distance. To account for bacterial density, we have normalized the pair-wise autocorrelation function to *g*_*rand*_*(r)* (Eq. (7)):

9 $${\Delta {\rm{g}}}_{N}=\frac{\Delta {\rm{g}}({\rm{r}})}{{{\rm{g}}}_{{rand}}(r)}=\frac{{\rm{g}}({\rm{r}})}{{{\rm{g}}}_{{rand}}(r)}-1$$

The function *Δg*_*N*_ describes the relative difference in the probability of finding a pair of bacteria separated by a distance r in a microscopy image compared to the probability expected for a spatially random distribution of the same number of bacteria. For example, if the bacteria are highly ordered in a microscopy image, like if they were continuously occurring at certain distance, r, then the value of *Δg*_*N*_ at this particular distance will be very high. The value of *Δg*_*N*_ = 1 at a particular distance r indicates that bacterial pairs are twice as likely to occur (i.e., a 100% increase) at that distance compared to a random distribution. In general, if bacteria are correlated in space, the autocorrelation function will have a positive value. Another example of images where one expects high values of *Δg*_*N*_ are the images with depicted bacterial aggregates. The bacteria in such an image are spatially highly correlated, giving high value of *Δg*_*N*_. More aggregates increase the value of *Δg*_*N*_ at the distances at or below the size of the aggregates.

The pair correlation analysis also allows estimation of the fraction of solitary bacteria—those without neighbors within a close range (e.g., ≤1.5 µm). This is calculated by counting all bacterial pairs within the distance threshold, dividing by two (to avoid double-counting), subtracting this number from the total number of bacteria in the image, and expressing the remainder as a percentage.

We used mosaic microscopy images of bacteria to obtain a representative large-scale bacterial distribution. The analyses were done on at least 5 independent samples, each consisting of at 9 view fields. Pair-wise correlations were done for at least 10^5^ pairs. A custom C++ computer code has been used to calculate autocorrelation.

### Kinetics of daptomycin activity on *Bacillus subtilis* native biofilms

After (15 ± 1) h of incubation in climatic chamber, biofilms were stained by adding 8 µL of PI solution (1 mM). After 30 min, 5 µL of daptomycin (30 mg/mL) or MSgp was applied on top of each biofilm grown on 8-well slides. The slides were then carefully covered with a glass coverslip without disturbing the biofilm surface and observed under an inverted microscope (Axio Observer Z1, LSM 800, Zeiss, Germany) using ZEN 2.6 software. To analyse biofilm physical structure and monitor the kinetics of daptomycin activity on *B. subtilis* biofilms, time-lapse confocal laser scanning module (LSM 800) was employed. The blue fluorescent protein (BFP) was excited using a diode laser at 405 nm (blue laser), while the PI was excited using a diode laser at 561 nm (red laser). The pinhole size was set to 1 AU, and the Z-axis sampling rate was set to 1 µm. Upper 12 µm of the biofilms were sampled. Scanning with the blue laser was performed at an intensity of 0.02–0.1%, and with the red laser at an intensity of 0.5–0.7%. At each time interval, emission light was recorded between 400 and 574 nm for BFP and 574–700 nm for PI using two GaAsP PMT detectors, operating at 850 V and 875 V, respectively. The image resolution was set to 1940 × 1940 pixels, as recommended by ZEN. Images were captured using a 40×/1.2 LD LCI Plan-Apochromat Imm Korr DIC M27 silicon oil objective. Observations were made on at least three independent biofilms. The counting of bacteria in the biofilm was done by a custom script written in ImageJ macro language. The script removes noise and background, determines independently the bacteria stained by PI and BFP and detects the bacteria colored by both dyes. PI enters only cells with disintegrated membranes, whereas BFP is constitutively expressed in all cells. PI-stained cells were counted as dead, while the total number of cells was determined as PI-stained + BFP colored cells.

### Data presentation and statistical analysis

For the statistical analysis of the data, we used SPSS (SPSS Inc., USA), Excel (Microsoft, USA) with Real statistics Add-On (www.real-statistics.com) and the online tools provided by GraphPad (https://www.graphpad.com/quickcalcs/ttest1/). Prior to statistical tests, the data representing ratios (e.g., cell survival), shear stress and yield stress were log-transformed to normalize distributions and stabilize variance. The data having at least six replicates were further tested for single outlier detection by performing Grubb’s test at *α* = 0.05. For multiple comparisons we used Welch’s ANOVA with Games-Howell post hoc analysis, except in the cases where the data was paired (native vs disintegrated biofilms). In the latter case we performed multiple paired *t*-tests with Holm-Bonferroni correction. For ordinal data obtained in MIC experiments we used pairwise Mann–Whitney *U* tests using exact test option, followed by Holm-Bonferroni correction. Two-tailed tests were always used. When comparing data curves for significance we calculated area under the curve (AUC) which was used in Welch’s ANOVA with Games-Howell post hoc analysis to determine statistical significance. In all statistical tests, samples showing *p* ≤ 0.05 were considered statistically significantly different; *p* ≤ 0.01 was considered highly significantly different and *p* ≤ 0.001 was considered extremely significantly different. Data presentation were performed in OriginPro (OriginLab, USA) program. Experimental errors indicated in text or graphs represent standard errors. Noise and background in the fluorescence microscopy images were removed using image processing software (Fiji/ImageJ or ZEISS ZEN). The images were afterwards pseudocolored: cyan for cells constitutively expressing BFP, red for propidium iodide (PI)-stained cells, and green for SYTO9-stained cells.DIC images were flat field corrected and contrast was enhanced.

## Supplementary information

Supplementary Information

## Data Availability

The authors declare that the data supporting the findings of this study are available within the paper and its Supplementary Material. Additional data are available from the corresponding author upon a reasonable request.

## Code availability

Custom C++ code for autocorrelation calculations and a custom ImageJ macro script for biofilm cell counting are available from the corresponding author upon reasonable request.
