# Supplementary material for: Biofilm structure as a key factor in antibiotic tolerance: insights from Bacillus subtilis model systems
Source: NPJ Biofilms Microbiomes. 2025 Dec 18;11:232. doi: 10.1038/s41522-025-00864-x (PMC12714748; doi:10.1038/s41522-025-00864-x)
Supplement: Supplementary file 1 — Supplementary Information [file 41522_2025_864_MOESM1_ESM.pdf]

Supplementary Information for

Biofilm Structure as a Key Factor in Antibiotic Tolerance: Insights from *Bacillus subtilis* Model Systems

Mojca Blaznik[1], Marko Volk[1], Barbara Kraigher[1], Alba Calonge-Sanz[1], Gema Barco García[1], David Stopar[1], Iztok Dogsa[1]*

1 Department of Microbiology, Biotechnical Faculty, University of Ljubljana, 1000, Ljubljana, Slovenia

*Corresponding author. Email: iztok.dogsa@bf.uni-lj.si

This file includes:
Supplementary Tables
Supplementary Figures
Supplementary References

SUPPLEMENTARY TABLES

Supplementary Table 1: The fraction of bacterial cells that appear solitary after biofilm disintegration and sample preparation for daptomycin exposure. For comparison also planktonic stationary culture is shown. The average of at least three biological replicates in corresponding standard deviation (SD) are shown.

CULTURE	% OF SOLITARY CELLS
**Planktonic wt**	96 ± 2
**Disintegrated wt**	81 ± 7
**Disintegrated Δ*tasA***	99 ± 1
**Disintegrated Δ*eps***	97 ± 1

Supplementary Table 2: Antimicrobial agents used in this study.

TARGET	ANTIMICROBIAL	PRODUCER	SOLVENT
**Protein synthesis**	Clindamycin	Supelco	MSgp
	Linezolid	Molekula	DMSO
	Gentamicin	Sigma - Aldrich	MSgp
	Spectinomycin	Alfa Aesar	MSgp
	Tetracycline	Sigma - Aldrich	MSgp
	Erythromycin	Carl Roth	MSgp
	Oxytetracycline	Sigma - Aldrich	MSgp
**Cell wall**	Fosfomycin	Apollo Scientific	MSgp
	Amoxicillin	Sigma - Aldrich	MSgp
	Ampicillin	Cayman Chemical	MSgp
	Vancomycin	Carl Roth	MSgp
**DNA**	Mitomycin C	Apollo Scientific	DMSO
	Ciprofloxacin	Thermo Fisher Scientific	DMSO + MSgp [1:3]
	PPI-102-2	1	DMSO
	AEC-609		
	PPI-13-3		
	END-85		
	AEC-271		
	AEC-154		
**Cell membrane**	CBG	Provided by ICANNA	DMSO + MSgp [1:3]
	Telavancin	Biosynth	DMSO
	Colistin	Cayman Chemical	MSgp
	Daptomycin	Thermo Fisher Scientific	MSgp/DMSO
	CBD	Provided by ICANNA	DMSO + MSgp [1:3]
**Cell wall + cell membrane**	Oritavancin	Biosynth	DMSO + MSgp [4:1]

SUPPLEMENTARY FIGURES

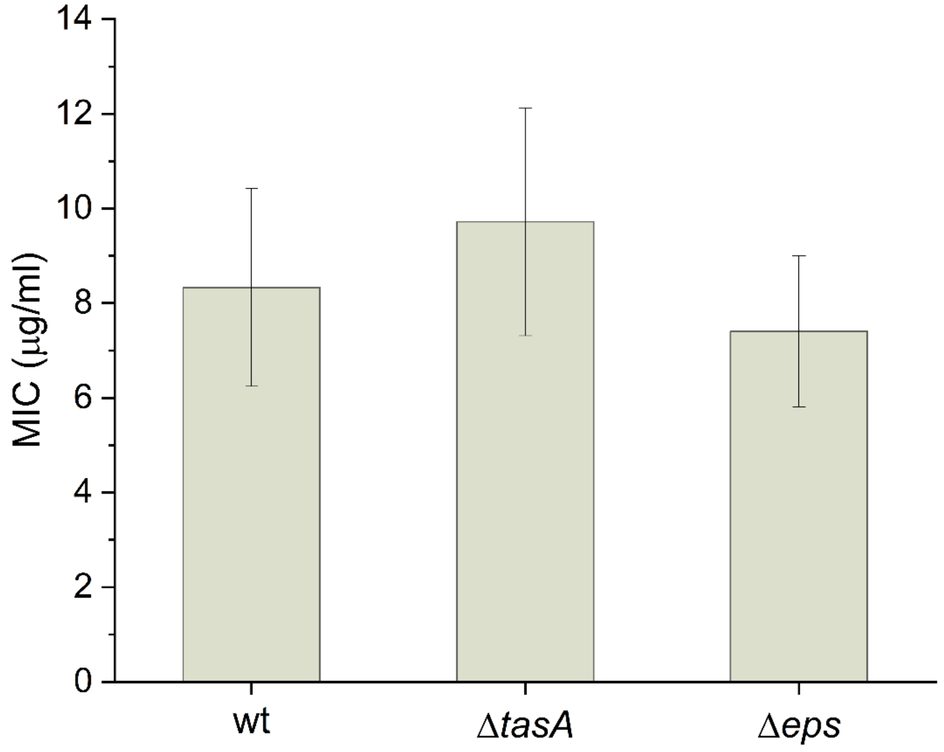

Supplementary Figure 1: Minimum inhibitory concentration (MIC) of daptomycin against different *Bacillus subtilis* strains grown as planktonic cultures. The differences were not statistically significant.

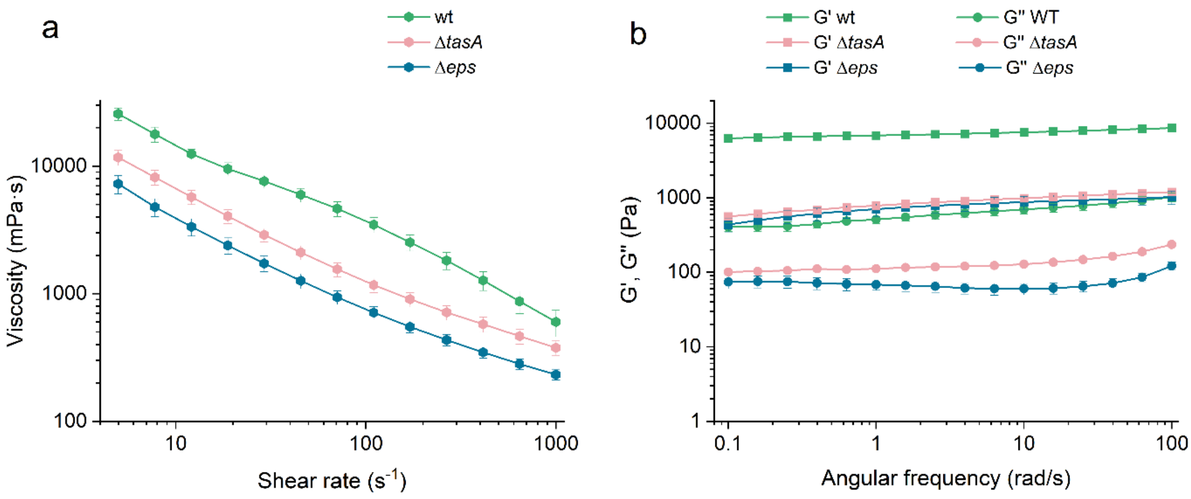

Supplementary Figure 2: Viscosity and viscoelastic properties of *B. subtilis* native biofilms in wt and corresponding mutants. **a** Viscosity measurements; **b** The storage G' and loss modulus G'' as a function of angular frequency. Results are presented as mean ± standard error from at least three independent biological measurements.

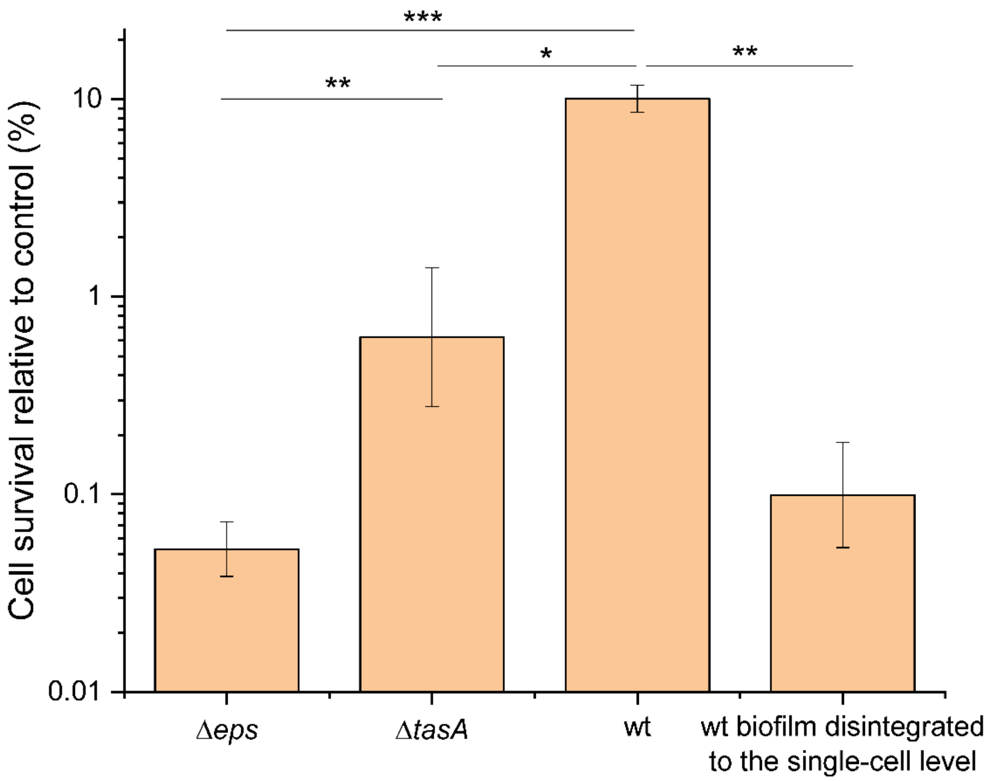

Supplementary Figure 3: Daptomycin efficacy in native *B. subtilis* biofilms of wild-type (wt), mutants, and in wild-type biofilms disintegrated to the single-cell level. Results were obtained using the spread plate method; the percentage of surviving cells in the antimicrobial-treated samples was normalized to that in the untreated controls. Data are presented as mean ± standard error from at least four independent biological replicates.; ns- not statistically significant, * - statistically significant, ** - highly significant, ***- extremely significant.

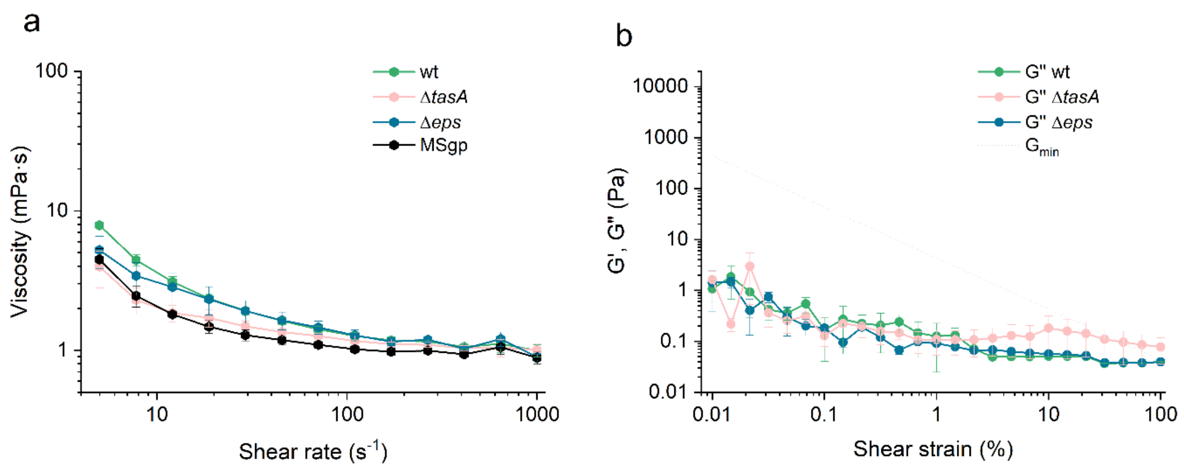

Supplementary Figure 4: Viscoelastic properties and viscosity of *B. subtilis* biofilms disintegrated to the single-cell level. **a** Viscosity measurements. **b** The loss modulus G" as a function of shear strain. Dotted line represents the boundary below which the values measured by the rheometer can become inaccurate. Results are presented as mean ± standard error from at least three independent biological measurements.

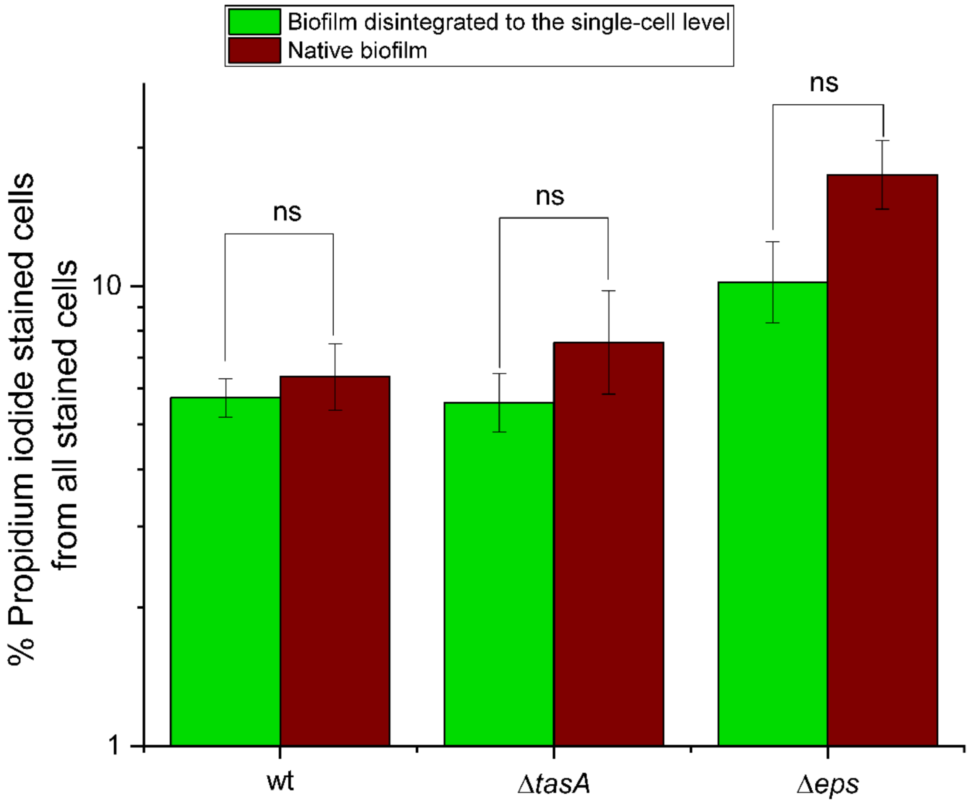

Supplementary Figure 5: The effect of the biofilm disintegration process (sonication) on fraction of propidium iodide (PI) stained *B. subtilis* cells. The biofilms cells were stained by SYTO9 that penetrates all the membranes regardless of their integrity and by PI that stains only cells with compromised membranes. Staining was performed after sonication. Data are presented as mean ± standard error from at least three independent biological replicates; ns- not statistically significant.

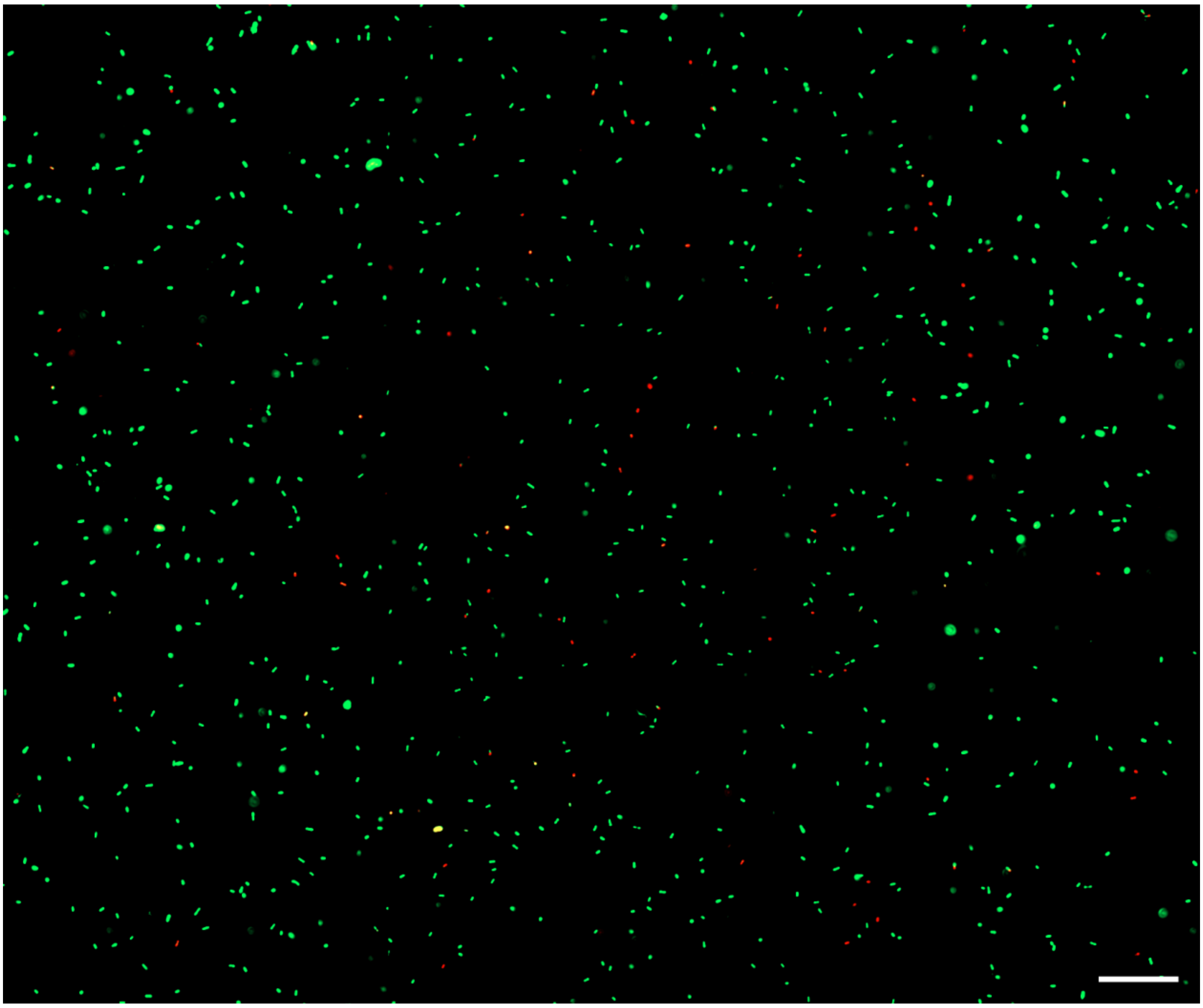

Supplementary Figure 6: Disintegration of the native wild-type *B. subtilis* biofilm to the single cell-level. For disintegration the sonication protocol as described in Material and methods section was used. The cells were stained after biofilm sonication by SYTO9 (green) and propidium iodide (red) and taken immediately for microscopic examination. The cells stained by propidium iodide were considered dead. The scale bar represents 10 µm.

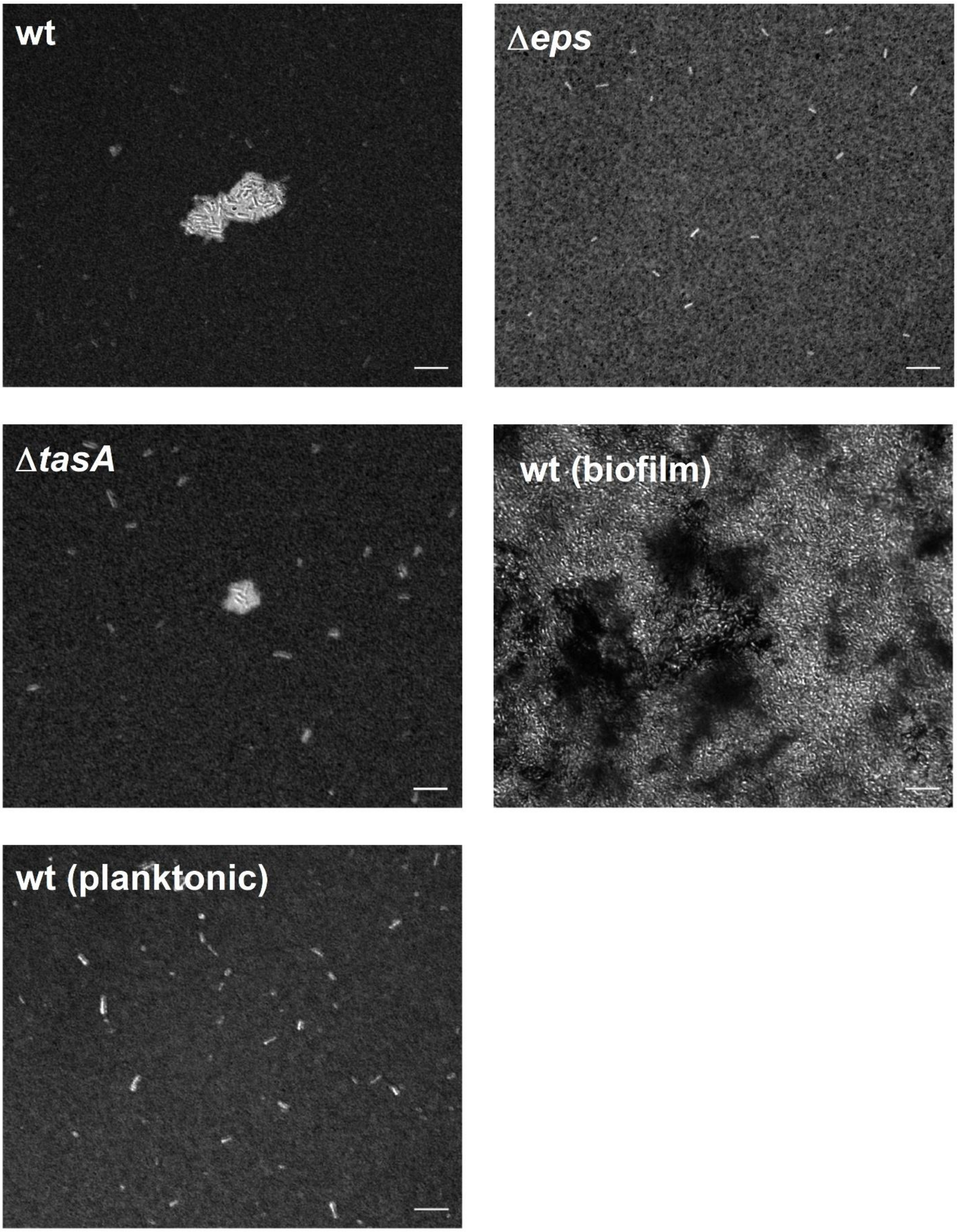

Supplementary Figure 7: Negative staining with Indian ink of disintegrated *B. subtilis* biofilms, native biofilm, and wild-type planktonic culture. Scale bar represents 10 µm.

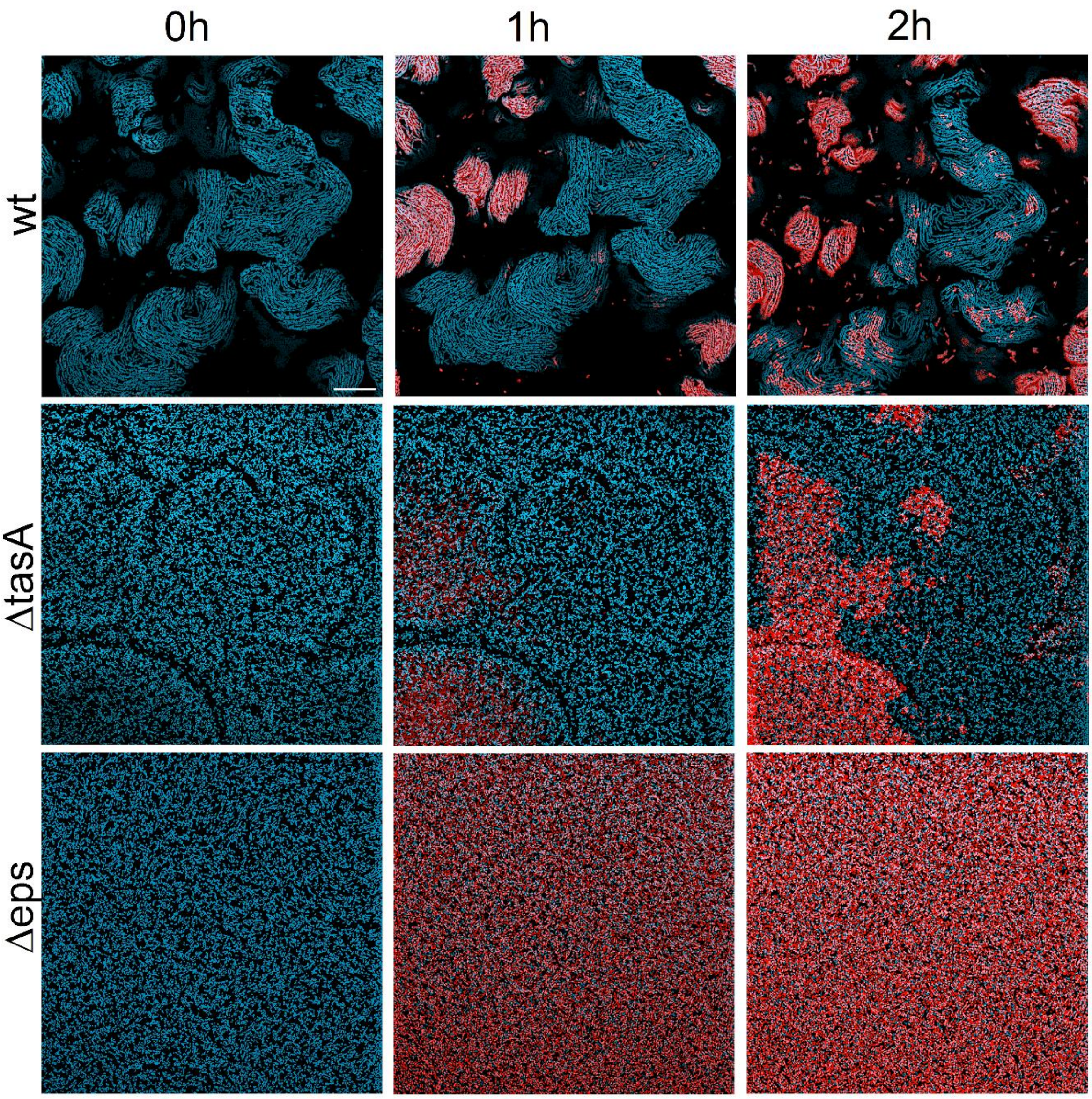

Supplementary Figure 8: The effect of extracellular biochemical structure on dynamics of daptomycin efficacy. Optical CLSM slices of native *B. subtilis* biofilms exposed to daptomycin, shown at 0 h, 1 h, and 2 h. All cells constitutively express blue fluorescent protein (BFP, shown in cyan), and cells with compromised membranes are stained with propidium iodide (PI, red). Scale bar: 20 μm.

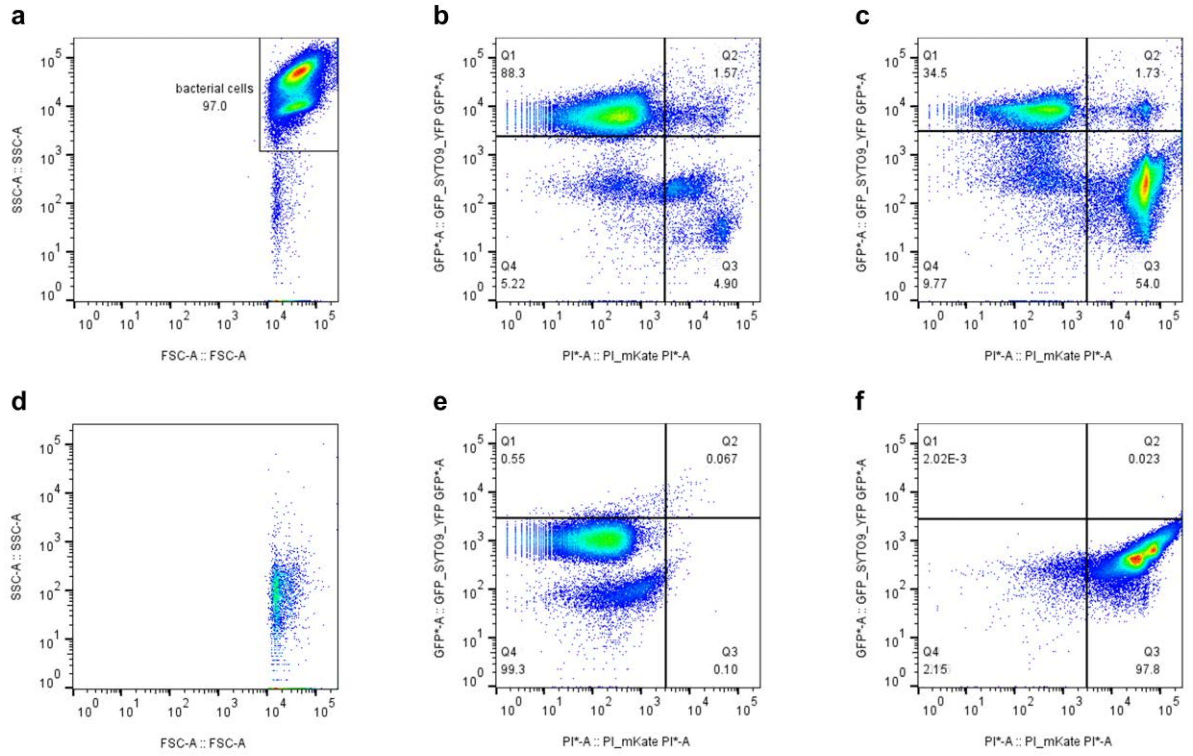

Supplementary Figure 9: Gating strategy for flow cytometry. **a** The distinction between noise and bacterial cells was made by gating FSC/SSC according to the noise present in the filtered MSgp sample. **b** Noise reference from the filtered MSgp sample. **c** Untreated *B. subtilis* biofilm sample, with alive cells identified by further gating on PI (propidium iodide) and SYTO 9 channels. **d** Daptomycin-treated biofilm sample, following the same gating strategy. **e** Unstained control used for gate setting. **f** Positive control (heated sample at 77 °C for 30 minutes) used for gate setting. The cells in Q1 are stained by the membrane-permeable DNA dye SYTO 9, indicating viability. The cells in Q2 and Q3 are stained by the membrane-impermeable DNA dye PI, indicating compromised membranes. The cells in Q4 are not stained by SYTO 9 or PI, indicating DNA loss due to cell lysis. Only the cells in Q1 were considered alive.

SUPPLEMENTARY REFERENCES

1. Cotman, A. E. *et al*. Discovery and Hit-to-Lead Optimization of Benzothiazole Scaffold-Based DNA Gyrase Inhibitors with Potent Activity against *Acinetobacter baumannii* and *Pseudomonas aeruginosa*. *J. Med. Chem.* **66**, 1380–1425 (2023).
